# CD147 antibody specifically and effectively inhibits infection and cytokine storm of SARS-CoV-2 and its variants delta, alpha, beta, and gamma

**DOI:** 10.1038/s41392-021-00760-8

**Published:** 2021-09-25

**Authors:** Jiejie Geng, Liang Chen, Yufeng Yuan, Ke Wang, Youchun Wang, Chuan Qin, Guizhen Wu, Ruo Chen, Zheng Zhang, Ding Wei, Peng Du, Jun Zhang, Peng Lin, Kui Zhang, Yongqiang Deng, Ke Xu, Jiangning Liu, Xiuxuan Sun, Ting Guo, Xu Yang, Jiao Wu, Jianli Jiang, Ling Li, Kun Zhang, Zhe Wang, Jing Zhang, Qingguo Yan, Hua Zhu, Zhaohui Zheng, Jinlin Miao, Xianghui Fu, Fengfan Yang, Xiaochun Chen, Hao Tang, Yang Zhang, Ying Shi, Yumeng Zhu, Zhuo Pei, Fei Huo, Xue Liang, Yatao Wang, Qingyi Wang, Wen Xie, Yirong Li, Mingyan Shi, Huijie Bian, Ping Zhu, Zhi-Nan Chen

**Affiliations:** 1 National Translational Science Center for Molecular Medicine & Department of Cell Biology, Fourth Military Medical University, Xi’an, 710032 China; 2 School of Medicine, Shanghai University, Shanghai, 200444 China; 3 Zhongnan Hospital of Wuhan University, Wuhan, 430071 China; 4 Division of HIV/AIDS and Sex-transmitted Virus Vaccines, Institute for Biological Product Control, National Institutes for Food and Drug Control (NIFDC) and WHO Collaborating Center for Standardization and Evaluation of Biologicals, Beijing, 102629 China; 5 Institute of Laboratory Animals Science, Chinese Academy of Medical Sciences, Beijing, 100071 China; 6 NHC Key Laboratory of Biosafety, National Institute for Viral Disease Control and Prevention, Chinese Center for Disease Control and Prevention, Beijing, 100871 China; 7 Beijing Institute of Biotechnology, Beijing, 100871 China; 8 Department of Clinical Immunology, Xijing Hospital, Fourth Military Medical University, Xi’an, 710032 China; 9 Beijing Institute of Microbiology and Epidemiology, Beijing, 100071 China; 10 School of Basic Medicine, Fourth Military Medical University, Xi’an, 710032 China; 11 Jiangsu Pacific Meinuoke Biopharmceutical Co. Ltd, Changzhou, 213022 China

**Keywords:** Infection, Inflammation

## Abstract

SARS-CoV-2 mutations contribute to increased viral transmissibility and immune escape, compromising the effectiveness of existing vaccines and neutralizing antibodies. An in-depth investigation on COVID-19 pathogenesis is urgently needed to develop a strategy against SARS-CoV-2 variants. Here, we identified CD147 as a universal receptor for SARS-CoV-2 and its variants. Meanwhile, Meplazeumab, a humanized anti-CD147 antibody, could block cellular entry of SARS-CoV-2 and its variants—alpha, beta, gamma, and delta, with inhibition rates of 68.7, 75.7, 52.1, 52.1, and 62.3% at 60 μg/ml, respectively. Furthermore, humanized CD147 transgenic mice were susceptible to SARS-CoV-2 and its two variants, alpha and beta. When infected, these mice developed exudative alveolar pneumonia, featured by immune responses involving alveoli-infiltrated macrophages, neutrophils, and lymphocytes and activation of IL-17 signaling pathway. Mechanistically, we proposed that severe COVID-19-related cytokine storm is induced by a “spike protein-CD147-CyPA signaling axis”: Infection of SARS-CoV-2 through CD147 initiated the JAK-STAT pathway, which further induced expression of cyclophilin A (CyPA); CyPA reciprocally bound to CD147 and triggered MAPK pathway. Consequently, the MAPK pathway regulated the expression of cytokines and chemokines, which promoted the development of cytokine storm. Importantly, Meplazumab could effectively inhibit viral entry and inflammation caused by SARS-CoV-2 and its variants. Therefore, our findings provided a new perspective for severe COVID-19-related pathogenesis. Furthermore, the validated universal receptor for SARS-CoV-2 and its variants can be targeted for COVID-19 treatment.

## Introduction

Globally emerged SARS-CoV-2 variants, such as alpha, beta, gamma, and delta, give rise to a new wave of epidemics. Delta, the most prevalent strain, was found in more than 130 countries. These variants are often characterized by shortened latency, increased transmissibility, increased risk of breakthrough infection, and reduced neutralizing antibody affinity postvaccination, which together led to increased morbidity globally.^1–4^ Mutations from SARS-CoV-2 are mostly found on the receptor-binding domain (RBD) of spike proteins, including N501Y, E484K, L452R, T478K, E484Q, which are crucial to pathogenicity.^5^ The rapid mutation also compromised the global effort for vaccination. Thus, it is urgent to develop a specific drug for SARS-CoV-2 variants, along with the global vaccination.

Severe COVID-19 cases were characterized by a cytokine storm characterized by elevated serum levels of cytokines and chemokines, including IL-1, IL-6, IL-8, IL-10, IL-17, CCL2, CXCL9, CXCL10, IFN-γ, and TNF-α.^6^ Cytokine storm is considered to be one of the major causes of acute respiratory distress syndrome (ARDS) and multipleorgan failure.^7^ It plays an important role in the process of disease aggravation, which results in prominent pathological features of COVID-19 pneumonia, featuring exudative diffuse alveolar damage with massive capillary congestion and hemorrhage, serous or fibrinous exudates, hyaline membrane formation, infiltration of macrophages, neutrophils, and lymphocytes, as well as lung consolidation.^8–10^ It is reported that the recruitment of Th17 cells and increased IL-17 caused cytokine storm, and type I IFN response exacerbated inflammation in severe COVID-19 cases.^11–13^ However, the underlying mechanism of cytokine storm in COVID-19 remains unclear.

CD147, an adhesion molecule, had been shown to be an important mediator of inflammatory and immune responses.^14^ CD147 can interact with cyclophilins and mediate the signaling and chemotactic activities of extracellular cyclophilin A (CyPA).^15–17^ Our previous study discovered that CD147 is a novel receptor for SARS-CoV-2 infection.^18^ Other laboratory also reported the involvement of CD147 in SARS-CoV-2 viral entry.^19^ However, whether CD147 can mediate cellular entry of SARS-CoV-2 variants remains unclear. In addition, whether CD147, as a signaling transducer, plays a role in the inflammation of COVID-19 diseases and contributes to cytokine storm in severe cases is unknown. Here, CD147 was found to be a universal receptor for SARS-CoV-2 and its variants, including alpha, beta, gamma, and delta. CD147 was also involved in the cytokine storm by regulating the expression of CyPA. In vivo experiments using a preclinical mouse model revealed that anti-CD147 antibody effectively inhibited the infection and cytokine storm of SARS-CoV-2 and its variants.

## Results

### CD147 antibody exhibits universal inhibition against SARS-CoV-2 and variants

**Fig. 1:**
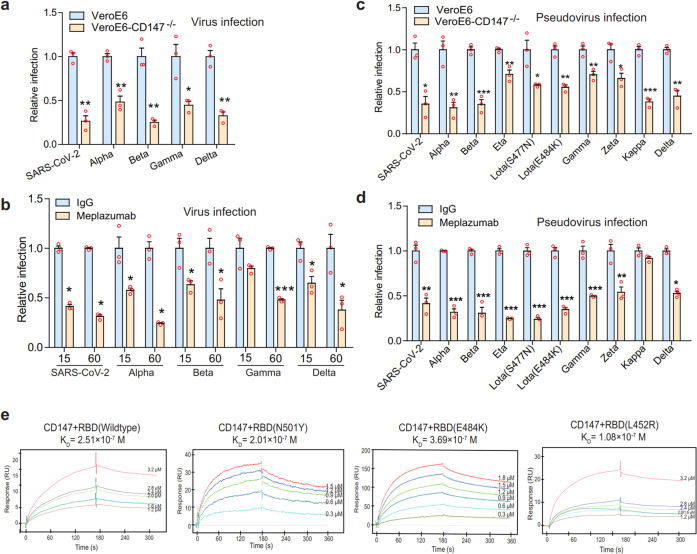
CD147 knockout and anti-CD147 antibody exhibit universal inhibition against SARS-CoV-2 and variants. **a** SARS-CoV-2 and its four variants—alpha, beta, gamma, and delta infected VeroE6 cells or CD147^−/−^ VeroE6 cells for 48 h, and RNA was collected for viral RNA detection by Taqman RT-qPCR. Data are normalized to the infectivity in wild-type VeroE6 cells (*n* = 3, two-tailed student *t* test, *p < 0.05, **p < 0.01). **b** Meplazumab inhibiting the infection of VeroE6 cells by SARS-CoV-2 and its four variants. Data are normalized to the infectivity in IgG group (*n* = 3, Two-tailed student *t* test, *p < 0.05, ***p < 0.001). **c** Viral infection efficiency was detected by luciferase reporter assay. VeroE6 cells and CD147^−/−^ VeroE6 cells were infected with the SARS-CoV-2 and nine mutant strains of SARS-CoV-2 pseudoviruses. Data are normalized to the infectivity in wild-type VeroE6 cells (*n* = 3, Two-tailed student *t* test, *p < 0.05, **p < 0.01, ***p < 0.001). **d** Meplazumab (60 μg/ml) inhibiting the infection of VeroE6 cells by SARS-CoV-2 and nine mutant strains of SARS-CoV-2 pseudoviruses. Data are normalized to the infectivity in IgG group (*n* = 3, Two-tailed student *t* test, *p < 0.05, **p < 0.01, ***p < 0.001). **e** The interaction of CD147 and RBD mu*t*ants detected by SPR assay

SARS-CoV-2 variants especially delta have been found in more than 100 countries, bringing challenges to control epidemics. To this end, we first investigated whether Meplazeumab, a humanized CD147 antibody, can block cellular entry of SARS-CoV-2 and its variants. CD147^−/−^ VeroE6 cell line was constructed and then infected with variants. The absence of CD147 decreased cellular entry of not only SARS-CoV-2 but also the four variants—alpha, beta, gamma, and delta significantly (Fig. 1a), with the reduced infection rates of 73.6, 51.7, 74.9, 55.3, and 67.4%, respectively (Table 1). Based on the concentration for 50% of maximal effect of Meplazumab calculated in our previous study,^18^ we used 15 and 60 μg/ml Meplazumab to inhibit the infection of SARS-CoV-2 and its four variants. At 15 μg/ml, the inhibition rates were 58.6, 42.4, 36.7, 20.3, and 35.2% respectively. At 60 μg/ml, the inhibition rates were 68.7, 75.7, 52.1, 52.1, and 62.3% respectively (Fig. 1b). We further infected nine mutant strains of pseudovirus, and found that infection was markedly suppressed by CD147 knockout (Fig. 1c and Table 2). Meplazumab effectively inhibited infection of all pseudovirus mutants except for kappa (Fig. 1d). The features of SARS-CoV-2, variants and SARS-CoV-2 pseudotyped variants and antibody inhibition rate were shown in Tables 3 and 4. The SARS-CoV-2 variants used in our study contain several mutation sites in RBD that were found in current prevalent variants, such as N501Y in alpha, beta, and gamma, E484K in beta and gamma, L452R in delta variants. The affinity constant between CD147 and RBD (wildtype) of SARS-CoV-2 was 2.51 × 10^-7^ M, comparable that the affinity constants between CD147 and RBD mutants N501Y, E484K, and L452R were 1.08 × 10^-7^ M, 1.01 × 10^-7^ M, and 3.69 × 10^-7^ M, respectively. The affinity ability between CD147 and RBD (wildtype) was not affected by the three RBD mutants (Fig. 1e), suggesting CD147 as a universal receptor mediates SARS-CoV-2 and its variants delta, alpha, beta, and gamma. Taken together, CD147 is a direct receptor for SARS-CoV-2 variants infection, and CD147 antibody exhibits universal inhibition against variants.

**Table 1:** SARS-CoV-2 variants features and the reduced infection rate in VeroE6-CD147^−/−^ cells

Virus name	Country of origin	Mutations (AA)	Reduced infection rate in VeroE6-CD147^−/−^ cells	Source
SARS-CoV-2	China		73.6%	National Institute for Viral Disease Control and Prevention, Chinese Center for Disease Control and Prevention
Alpha	United Kingdom	HV69-70 Del, Y145 Del, N501Y, A570D, D614G, P681H, T716I, S982A, D1118H	51.7%	
Beta	South Africa	D80A, D215G, LAL242–244 Del, E484K, K417N, N501Y, D614G, A701V	74.9%	
Gamma	Brazil	L18F, T20N, P26S, D138Y, R190S, K417T, E484K, N501Y, D614G, H655Y, T1027I, V1176F	55.3%	
Delta	India	T19R, T95I, G142D, L452R, T478K, D614G, P681R, D950N, R158del	67.4%

**Table 2:** SARS-CoV-2 pseudotyped variants features and the reduced infection rate in VeroE6-CD147^−/−^ cells

Pseudotyped virus name	Country of origin	Mutations (AA)	Reduced infection rate in VeroE6-CD147^−/−^ cells	Source
SARS-CoV-2	China		64.6%	National Institutes for Food and Drug Control, China
Alpha	United Kingdom	HV69-70 Del, Y145 Del, N501Y, A570D, D614G, P681H, T716I, S982A, D1118H	69.1%	
Beta	South Africa	D80A, D215G, LAL242–244 Del, E484K, K417N, N501Y, D614G, A701V	64.9%	
Eta	Denmark, America	Q52R, A67V, H69del, V70del, Y144del, E484K, D614G, Q677H, F888L	29.2%	
Lota (S477N)	America	L5F, T95I, D253G, S477N, D614G, Q957R	42.0%	
Lota (E484K)	America	L5F, T95I, D253G, E484K, D614G, A701V	44.7%	
Gamma	Brazil	L18F, T20N, P26S, D138Y, R190S, K417T, E484K, N501Y, D614G, H655Y, T1027I, V1176F	30.0%	
Zeta	Brazil	E484K, D614G, V1176F	34.0%	
Kappa	India	T951, G142D, E154K, L452R, E484Q, D614G, P681R, Q1071H	62.0%	
Delta	India	T19R, G142D, F157del, L452R, T478K, D614G, P681R, D950N	55.2%

**Table 3:** SARS-CoV-2 variants features and CD147 antibody inhibition rate

Virus name	Country of origin	Mutations (AA)	Meplazumab inhibition rate (60 μg/ml)	Source
SARS-CoV-2	China		68.7%	National Institute for Viral Disease Control and Prevention, Chinese Center for Disease Control and Prevention
Alpha	United Kingdom	HV69-70 Del, Y145 Del, N501Y, A570D, D614G, P681H, T716I, S982A, D1118H	75.7%	
Beta	South Africa	D80A, D215G, LAL242–244 Del, E484K, K417N, N501Y, D614G, A701V	52.1%	
Gamma	Brazil	L18F, T20N, P26S, D138Y, R190S, K417T, E484K, N501Y, D614G, H655Y, T1027I, V1176F	52.1%	
Delta	India	T19R, T95I, G142D, L452R, T478K, D614G, P681R, D950N, R158del	62.3%

**Table 4:** SARS-CoV-2 pseudotyped variants features and CD147 antibody inhibition rate

Pseudotyped virus name	Country of origin	Mutations (AA)	Meplazumab inhibition rate (60 μg/ml)	Source
SARS-CoV-2	China		58.7%	National Institutes for Food and Drug Control, China
Alpha	United Kingdom	HV69-70 Del, Y145 Del, N501Y, A570D, D614G, P681H, T716I, S982A, D1118H	68.2%	
Beta	South Africa	D80A, D215G, LAL242–244 Del, E484K, K417N, N501Y, D614G, A701V	69.4%	
Eta	Denmark, America	Q52R, A67V, H69del, V70del, Y144del, E484K, D614G, Q677H, F888L	75.3%	
Lota (S477N)	America	L5F, T95I, D253G, S477N, D614G, Q957R	76.0%	
Lota (E484K)	America	L5F, T95I, D253G, E484K, D614G, A701V	65.2%	
Gamma	Brazil	L18F, T20N, P26S, D138Y, R190S, K417T, E484K, N501Y, D614G, H655Y, T1027I, V1176F	50.3%	
Zeta	Brazil	E484K, D614G, V1176F	46.0%	
Kappa	India	T951, G142D, E154K, L452R, E484Q, D614G, P681R, Q1071H	8.37%	
Delta	India	T19R, G142D, F157del, L452R, T478K, D614G, P681R, D950N	47.3%	

### Humanized CD147 (hCD147) transgenic mouse model with SARS-CoV-2 and variants infection mimics COVID-19 pathology

**Fig. 2:**
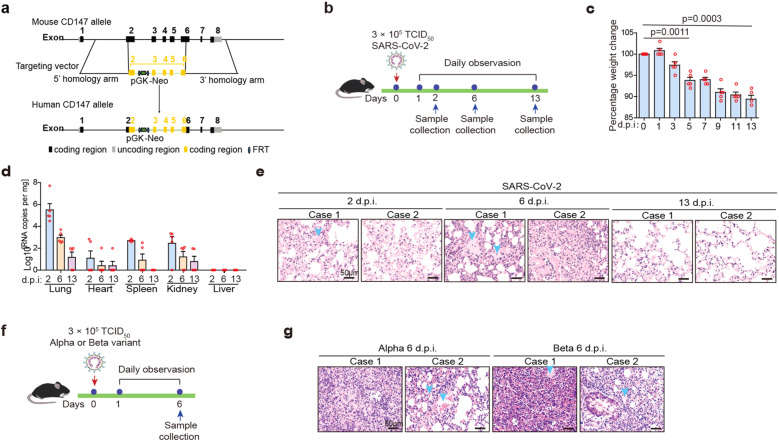
Humanized CD147 transgenic mouse model with SARS-CoV-2 and variants infection mimics COVID-19 pathology. **a** Schematic diagram of the method to construct hCD147 mice. **b** Inoculation of hCD147 mice via the intranasal at 3 × 10^5^ TCID_50_ of SARS-CoV-2, and sample collected at 2, 6, and 13 d.p.i. **c** Weight changes were monitored (*n* = 5). **d** Taqman RT-qPCR for viral RNA levels in the lung, heart, spleen, kidney, and liver (*n* = 5). **e** H&E staining of lung tissue sections from infected hCD147 mice (scale bars, 50 μm). **f** hCD147 mice were inoculated via the intranasal route with 3 × 10^5^ TCID_50_ of alpha or beta variant, and sample collected at 6 d.p.i. **g** H&E staining of lung tissue sections from infected hCD147 mice (scale bars, 50 μm)

Severe COVID-19 patients present features of exudative alveolar pneumonia with endocrine and immune disorders, such as inappropriate adrenal response to stress, subacute thyroiditis, hyperglycemia, and hyperinflammation.^20,21^ Further studies are urgently needed to establish the extent and mechanisms of how COVID-19 affects endocrine and immune systems. In our study, a human CD147 (hCD147) transgenic mouse model was constructed by replacing mouse CD147 ectodomain with human’s in embryonic stem cells (Fig. 2a and Supplementary Fig. 1a, b). We then inoculated hCD147 mice via the intranasal route with 3 × 10^5^ TCID_50_ of SARS-CoV-2 (Fig. 2b). After 5 days postinfection (d.p.i.), hCD147 mice demonstrated marked weight loss, and all mice lost approximately 10% of their body weight by 13 d.p.i. (Fig. 2c). High levels of viral RNA were detected in lung homogenates at 2 d.p.i., and in some of the mice, low levels of viral RNA were detected in organs, such as the heart, spleen, and kidney (Fig. 2d). H&E-stained lung sections from virus-infected hCD147 mice showed distinctive pathological characteristics at three-time points, with the most severe symptoms at 6 d.p.i., characterized by desquamating of alveolar epithelial cells, serofluid exudation, infiltration of macrophages, neutrophils and lymphocytes, hemorrhage, and pulmonary consolidation (Fig. 2e and Supplementary Fig. 1c). Meanwhile, we inoculated hCD147 mice with alpha and beta variants (Fig. 2f), which caused similar lung pathological changes to SARS-CoV-2 (Fig. 2g and Supplementary Fig. 1d). All these lung pathological changes were consistent with COVID-19 patients.

### hCD147 mouse model with SARS-CoV-2 infection showed similar immune characteristics to those of COVID-19 patients

**Fig. 3:**
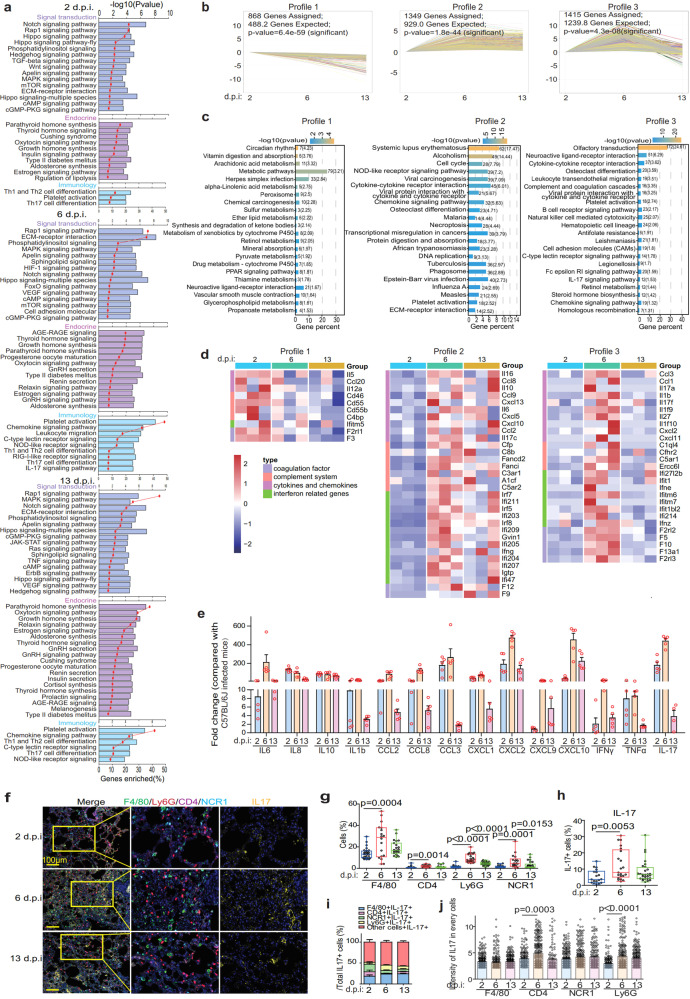
Immune characteristics of hCD147 mouse model with SARS-CoV-2 infection. **a** RNA-seq analysis of the lung homogenates of C57BL/6 J and hCD147 mice with SARS-CoV-2 infection at 2, 6, and 13 d.p.i. (*n* = 3). KEGG enrichment analysis of pathways enriched in differentially expressed genes. **b** The statistically significant profiles by trend analysis of differentially expressed genes at 2, 6, and 13 d.p.i. **c** KEGG enrichment analysis of pathways enriched in the three profiles. **d** Heatmap of significantly upregulated genes in the three profiles. Columns represent samples and rows represent genes. Gene expression levels in the heat maps are *z* score–normalized values determined by log_2_^[CPM]^ values. **e** Fold change in the gene expression of the cytokines and chemokines determined by RT-qPCR, compared with C57BL/6 J controls in lung homogenates at 2, 6, and 13 d.p.i. Gapdh is used as a reference gene (*n* = 5). **f** Multiplex immunofluorescence staining of lung tissue sections for F4/80, CD4, Ly6G, NCR1, and IL-17 from hCD147 mice at 2, 6, and 13 d.p.i. (scale bars, 50 μm). **g** Statistics of the percentage of cells positive for F4/80, CD4, Ly6G, and NCR1 (Two-tailed student *t* test, each dot represents an image). **h** Statistics of the percentage of cells positive for IL-17 (Two-tailed student *t* test, each dot represents an image). **i** The composition of IL-17 positive cells in lung tissues at 2, 6, and 13 d.p.i. **j** The intensity of IL-17 on macrophages, CD4 + T cells, neutrophiles, and NK cells in lung tissues. Each dot represents a cell

To further investigate the pathogenesis of virus-infected hCD147 mouse model, RNA-seq sequencing was performed on the lung tissues of SARS-CoV-2-infected mice at different time points (Supplementary Fig. 2a). Kyoto Encyclopedia of Genes and Genomes (KEGG) enrichment showed that SARS-CoV-2-activated multiple signal transduction signalings involved in the lung injury (cAMP signaling pathway) and inflammation (MAPK, JAK and mTOR signaling pathways)^22,23^ in hCD147 mice at 2, 6, and 13 d.p.i. (Fig. 3a). As well, the endocrine system signalings (thyroid hormone signaling) and immunology signalings (platelet activation signal; Th1, Th2, and Th17 cells differentiation) were observed in infected hCD147 mice (Fig. 3a), which were also observed in COVID-19 patients.^24–26^ Then, we conducted trend analysis of the RNA-seq data to enrich genes with three different profiles, including profile 1 (continuously decreasing from 2 to 13 d.p.i.), profile 2 (reaching the plateau at 6 d.p.i.), and profile 3 (reaching the peak at 6 d.p.i.) (Fig. 3b). Multiple metabolism-related pathways continuously decreased from 2 to 13 d.p.i., while immune-related signals reached the peak at 6 d.p.i., including the interaction between the cytokines and cytokine receptors, IL-17 signaling, cytokines and chemokine signalings, platelet activation, B cell receptor signaling, leukocyte migration, NK cell-mediated cytotoxicity and complement and coagulation response (Fig. 3c). A large number of cytokines and chemokines (IL-6, CCL2, IL-17, IL1B, IL10, CCL8, CXCL10, etc.), complement system (C8b, C3ar1, C1q14, etc.), and coagulation factors (F12, F9, F10, F5, etc.) reached the highest level at 6 d.p.i. (Fig. 3d). RT-qPCR verified several elevated cytokines and chemokines in the lung tissues of SARS-CoV-2-infected hCD147 mice, compared with that of virus-infected C57BL/6 J mice (Fig. 3e). All these results suggested that SARS-CoV-2 could induce endocrine dyscrasia and strong immune responses through CD147.

Th17 cell response was reported crucial in the initiation of cytokine storm in COVID-19.^27^ We focused on analyzing the IL-17 expression on macrophages, CD4 + cells, neutrophiles, and NK cells using multiplex immunofluorescence (Fig. 3f and Supplementary Fig. 2b). Both the number of inflammatory cells and the level of IL-17 expression reached the maximum at 6 d.p.i. (Fig. 3g, h). IL-17 was found expressed on macrophages, CD4 + T cells, neutrophils, and NK cells. In all IL-17 positive cell population, the proportion of neutrophils increased significantly at 6 d.p.i. (Fig. 3i); CD4 + T cells and neutrophils showed increased IL-17 intensity at 6 d.p.i. (Fig. 3j), indicating that the enhanced IL-17 response was mainly attribute to the involvement of CD4 + T cells and neutrophils. We then analyzed the expression of IL-17 in the lung tissues of COVID-19 patients (Supplementary Fig. 2c), and the results showed that the composition of IL-17+ cells and the intensity of IL-17 were consistent with that of infected hCD147 mice at 6 d.p.i. (Supplementary Fig. 2d, e), which further reflects that hCD147 mice well match the characteristics of COVID-19 patients, and CD147 might contribute to cytokine storm of COVID-19. To summarize, these results confirmed that hCD147 mice could well simulate pathological manifestations and immune characteristics of COVID-19 patients, indicating that CD147 plays an important role in the pathogenesis and cytokine storm induced by SARS-CoV-2 infection, and hCD147 mouse is an ideal model for studying the pathogenesis of COVID-19.

### SARS-CoV-2 causes different pneumonia phenotypes and immune responses in the hACE2 and hCD147 mouse models

**Fig. 4:**
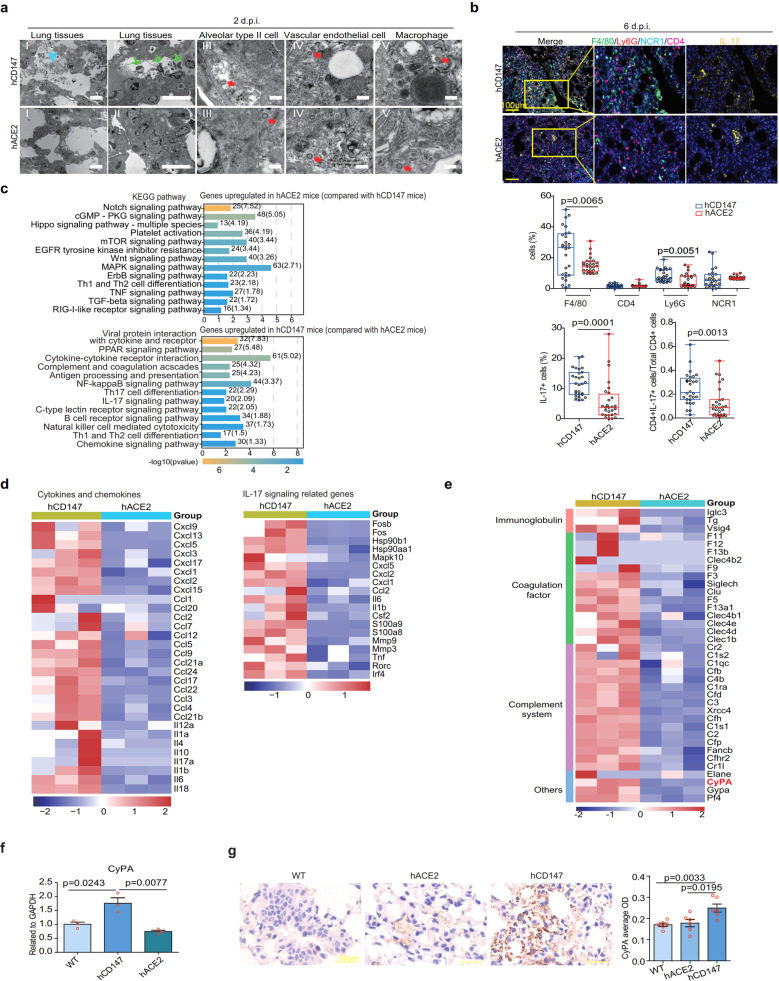
SARS-CoV-2 causes exudative alveolar pneumonia phenotypes and stronger immune responses in hCD147 mouse model compared with hACE2 mouse model. hACE2 and hCD147 mice were inoculated via the intranasal route with 3 × 10^5^ TCID_50_ of SARS-CoV-2. Lung tissues were collected at 2, 6, and 13 d.p.i. for pathological analysis. **a** Pathological changes in hACE2 and hCD147 mouse lung tissues (at 2 d.p.i.) were detected by electron microscopy (scale bars in panels I and II, 10 μm; scale bars in panels III–V, 200 nm). The cyan arrow indicates exudation; the green arrows indicate infiltration of macrophages, neutrophils, and erythrocytes; the red arrows indicate virions. **b** Multiplex immunofluorescence staining for CD4, F4/80, Ly6G, NCR1, and IL-17 at 6 d.p.i. by SARS-CoV-2, and statistics of the percentages of cells positive for CD4, F4/80, Ly6G, NCR1, and IL-17 (Each dot represents an image, Two-tailed student *t* test). **c** KEGG enrichment analysis of pathways enriched in upregulated differentially expressed genes in SARS-CoV-2-infected hACE2 mice and hCD147 mice at 2 d.p.i. **d** Heatmap of significantly upregulated cytokines, chemokines, IL-17 signaling-related genes in lung homogenates of SARS-CoV-2-infected hCD147 mice compared with SARS-CoV-2-infected hACE2 mice. **e** Heatmap of immune-related genes (except cytokines and chemokines) in lung homogenates of SARS-CoV-2-infected hCD147 mice compared with SARS-CoV-2-infected hACE2 mice. Columns represent samples and rows represent genes. Gene expression levels in the heat maps are z score–normalized values determined by log_2_^[CPM]^ values. **f** RT-qPCR for CyPA levels in lung homogenates of SARS-CoV-2-infected C57BL/6 J, hACE2, and hCD147 mice (*n* = 3, two-tailed unpaired *t* test). **g** Detection of CyPA expression in lung tissues from SARS-CoV-2-infected C57BL/6 J, hACE2, and hCD147 mice by immunohistochemical staining

Though cytokine storm is closely related with COVID-19 progress, regulation of cytokine storm by virus infection remains unclear. We first compared pathology and inflammation features between the hACE2^28^ and hCD147 mouse models infected with SARS-CoV-2. The pneumonia phenotype reached the peak at 6 d.p.i. in both the models (Supplementary Fig. 3a). Notably, hACE2 mice presented interstitial pneumonia, whereas hCD147 mice showed exudative alveolar inflammation with more exudation and alveolar wall damage (Supplementary Fig. 3a). Under the electron microscope, the infiltration of macrophages and neutrophils, leakage of erythrocytes and exfoliated alveolar type II epithelial cells were observed in alveoli of hCD147 mice, whereas these cells were rarely observed in alveoli of hACE2 mice at 2 d.p.i. (Fig. 4a). At 6 d.p.i., more macrophages and neutrophils were found in the lung tissues of hCD147 mice than hACE2 mice. However, the number of CD4 + T cells and NK cells were comparable (Fig. 4b). Notably, the number of IL-17+ cells significantly increased in the lung tissues of hCD147 mice (Fig. 4b). Although the proportion of CD4 + T cells was similar in both mice, the proportion of CD4 + IL-17 + T cells was significantly higher in hCD147 mice (Fig. 4b). All the results indicate that SARS-CoV-2 infection via CD147 induces stronger innate and adaptive immune responses, which may explain the decreased viral titer in lung tissues of hCD147 mouse at 6 d.p.i. Based on the observed different pneumonia phenotypes between SARS-CoV-2-infected hCD147 and hACE2 mouse models at 2 d.p.i., we assume a different immune response between the two mouse models at the initiation of virus infection. To prove the hypothesis, we performed RNA-seq using mouse lung tissues at 2 d.p.i. (Supplementary Fig. 3b). KEGG analysis showed that SARS-CoV-2-activated NF-κB, IL-17, B cell receptors, and cytokine/chemokine-related signaling pathways in hCD147 mice that more likely triggered the cytokine storm, while it activated Notch, MAPK, TNF, and TGFβ signaling pathways in hACE2 mice (Fig. 4c). When compared with the hACE2 mouse model, the differentially upregulated genes in SARS-CoV-2-infected hCD147 mice were enriched in many cytokines and chemokines, as well as IL-17 signaling-related genes (Fig. 4d). These results indicate that SARS-CoV-2 causes different immune responses through CD147 and ACE2 receptors, and that CD147-mediated proinflammatory responses are stronger, which may trigger the cytokine storm.

To make clear the molecular mechanism, we analyzed the upregulated immune-related genes in hCD147 infected mice, compared with hACE2 mouse model. In addition to cytokines and chemokines, the highly expressed genes related to immune system were immunoglobulin, coagulation factor, and complement system (Fig. 4e). It is worth-noticing that CyPA, as an upstream regulator of effectors, such as IL-6, IL1b, IL1a, CXCL1, and CXCL2,^29^ showed significant upregulation in hCD147 mice (Fig. 4e), which was verified by RT-qPCR and immunohistochemical staining (Fig. 4f, g), inferring that CyPA may be the key intermediate proinflammatory factor in CD147-related immune responses upon SARS-CoV-2 infection.

### Spike protein-CD147-CyPA axis contributes to COVID-19 cytokine storm

**Fig. 5:**
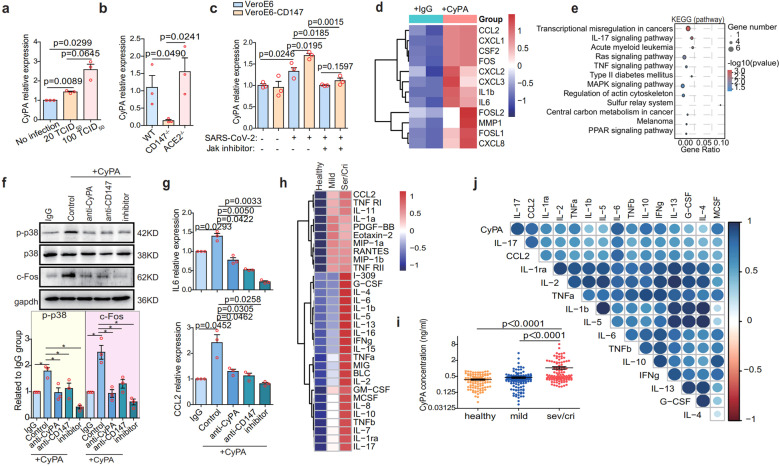
Spike protein-CD147-CyPA axis contributes to COVID-19 cytokine storm. **a** SARS-CoV-2-infected VeroE6 cells with different titers were incubated for 48 h, and RNA was collected for CyPA detection by RT-qPCR (*n* = 3, Two-tailed student *t* test). **b** CD147 or ACE2 in VeroE6 cells was knocked out by CRISPR-Cas9, and the cells were then infected with SARS-CoV-2 for 48 h. The expression level of CyPA was detected by RT-qPCR (*n* = 3, Two-tailed student *t* test). **c** CD147 in VeroE6 cells was ectopically expressed by lentivirus, the cells were then infected with SARS-CoV-2 for 48 h with or without JAK inhibitor. The expression level of CyPA was detected by RT-qPCR (*n* = 3, Two-tailed student *t* test). **d** Heatmap of significantly upregulated genes based on RNA-seq. BEAS-2B cells were stimulated with or without CyPA for 48 h. Columns represent samples and rows represent genes. Gene expression levels in the heat maps are *z* score–normalized values determined by log_2_^[CPM]^ values. **e** KEGG enrichment analysis of pathways enriched in upregulated genes from the CyPA-stimulated group compared with the control group. **f** Western blot analysis of p-p38, p-38, and c-FOS expression (*n* = 3, Two-tailed student *t* test, *p < 0.05). **g** IL-6 and CCL2 expression detected by RT-qPCR. **h** The heatmap of 32 differential cytokines. Fourty human cytokines were detected in plasma from healthy individuals (*n* = 100) and COVID-19 patients (*n* = 200, including 41 severe/critical and 159 mild) using cytokine chips. **i** CyPA prote**i**n detected in the plasma of healthy donors (*n* = 100), COVID-19 patients with mild symptoms (*n* = 159) and COVID-19 patients with severe/critical symptoms (*n* = 106) by ELISA (Two-tailed student *t* test). **j** Correlation analysis between CyPA and cytokines according to the Pearson correlation coefficient. Data were collected from the 41 severe/critical cases

To further determine that CyPA is a key intermediate proinflammatory factor that promotes the pathological process of SARS-CoV-2 infection via CD147, we conducted experiments in cell lines. In VeroE6 cells, upregulation of CyPA was induced by different virus titer (Fig. 5a). When VeroE6 cells were infected with SARS-CoV-2, alpha and beta variants, CD147 knockout, rather than ACE2 knockout, significantly reduced CyPA expression (Fig. 5b and Supplementary Fig. 4a, b). Meanwhile, SARS-CoV-2 induced CyPA expression was enhanced by CD147 overexpression, which was inhibited by JAK inhibitor (Fig. 5c), indicating that CD147 is responsible for CyPA upregulation via JAK pathway.^30^

CyPA is a ubiquitously expressed intracellular protein and is secreted in response to inflammatory stimuli.^31^ Therefore, CyPA antibody was used to neutralize the soluble CyPA to evaluate whether the expression of cytokines and chemokines would be affected. Evidently, the CyPA antibody reduced the expression of IL-6 and CCL2 (Supplementary Fig. 4d), with the viral load unchanged (Supplementary Fig. 4c). To map the CyPA-related transcriptome networks, RNA-seq was performed using human lung epithelial BEAS-2B cells, which were stimulated with CyPA. In consistent with the in vivo data from SARS-CoV-2-infected hCD147 mice (Fig. 4d), CyPA-stimulated BEAS-2B cells showed upregulated expression of a variety of proinflammatory cytokines and chemokines, including IL-6, IL-1b, CCL2, CXCL1, and CXCL2, as well as several AP-1 family members (c-FOS, FOSL1, and FOSL2) (Fig. 5d) that promote the transcription of multiple cytokine genes during inflammation.^32^ KEGG enrichment showed that CyPA stimulation significantly upregulated IL-17 signaling (Fig. 5e), which was also active in infected hCD147 mice. Meanwhile, CyPA-activated MAPK pathway which can regulate AP-1,^33^ suggesting that CyPA regulates cytokine expression through the MAPK-AP-1 pathway. We proved that CyPA induced MAPK-AP-1 pathway could be inhibited by MAPK inhibitor, reversing the increase of p-p38 and c-FOS expression (Fig. 5f). As we all know, CD147 is a major receptor for extracellular CyPA.^34–36^ Co-IP and SPR analysis showed the interaction between CD147 and CyPA with an affinity constant K_D_ of 3.34 × 10^-8^ M (Supplementary Fig. 4e, f). CD147 antibody could also reverse the increase of p-p38 and c-FOS expression induced by CyPA, the same as CyPA antibody (Fig. 5f). In addition, the expression of IL-6 and CCL2 was effectively inhibited by the above three agents (Fig. 5g). These results demonstrated that CyPA induces cytokine expression through the CD147-mediated MAPK pathway.

To confirm the clinical significance of CyPA in the inflammatory responses in COVID-19, a cytokine chip assay was performed and showed that 32 out of 40 cytokines were upregulated in plasma of COVID-19 patients (*n* = 200, including 41 severe/critical and 159 mild), compared with healthy individuals (*n* = 100) (Fig. 5h and Supplementary Fig. 5). Meanwhile, CyPA level in plasma analyzed by ELISA was significantly higher in severe/critical COVID-19 patients (*n* = 106, comprising the above 41 severe/critical cases) than in mild (*n* = 159) and healthy cohorts (*n* = 100) (Fig. 5i). Further, CyPA level in clinical samples was significantly correlated with those above upregulated cytokine level, especially IL-17, CCL2, and IL-6 (Fig. 5j). Then we detected the expression of CyPA, macrophage marker CD68, CCL2, and IL-6 in lung tissues from 13 cases of COVID-19 patients using multiplex immunofluorescence (Supplementary Fig. 6a). Each collected image was divided into nine process regions, and the mean fluorescence intensity (MFI) was analyzed with Halo software and correlation analysis was performed (Supplementary Fig. 6b). The results showed that in the lung tissues of patients with COVID-19, CyPA had strong positive correlations with CD68, CCL2, and IL-6 (Supplementary Fig. 6c, d). In particular, the correlation coefficient reached 0.8 between the CyPA and CD68, indicating that CyPA is related to the infiltration of macrophages. These results indicated that CyPA is involved in inflammatory responses in COVID-19. Furthermore, CyPA expression was significantly elevated in CD147^high^ regions than CD147^low^ regions, and CD147 expression had a strong positive correlation with CyPA (Supplementary Fig. 6e), which further verified that CD147 upregulated CyPA expression upon SARS-CoV-2 infection. So, we confirmed that CyPA is a key intermediate proinflammatory factor involved in CD147-related immune responses upon SARS-CoV-2 infection. Spike protein of SARS-CoV-2 bound to CD147 and initiated the JAK-STAT pathway, which induced expression of CyPA. CyPA reciprocally bound to CD147, triggered MAPK pathway and consequently mediated the expression of cytokines and chemokines.

### CD147 antibody reverses pulmonary inflammation caused by SARS-CoV-2 and variants alpha and beta

**Fig. 6:**
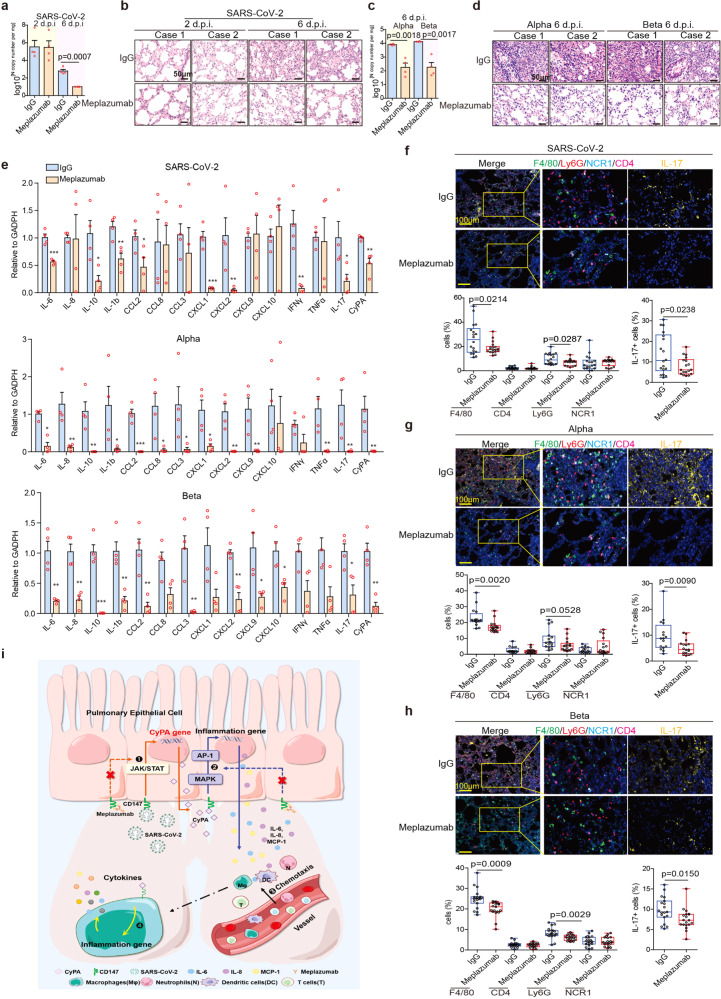
Anti-CD147 antibody reverses pulmonary inflammation caused by SARS-CoV-2 and its variants. hCD147 mice were intranasally inoculated with 3 × 10^5^ TCID50 SARS-CoV-2, alpha or beta variant and treated with anti-CD147 antibody Meplazumab the next day. Lung tissues were collected for analysis. **a** RT-qPCR for viral RNA levels in lung tissues at 2 and 6 d.p.i (*n* = 4). **b** H&E staining of lung tissue sections from the IgG and Meplazumab groups at 2 and 6 d.p.i (scale bars, 50 μm). **c** RT-qPCR for viral RNA levels in lung tissues at 6 d.p.i by alpha or beta variant (*n* = 4). **d** H&E staining of lung tissue sections from the IgG and Meplazumab groups at 6 d.p.i by alpha or beta variant (scale bars, 50 μm). **e** Gene expression of cytokines and chemokines in lung homogenates determined by RT-qPCR, compared with the corresponding IgG controls at 6 d.p.i. by SARS-CoV-2, alpha or beta variant (*n* = 4, Two-tailed student *t* test, *p < 0.05). Gapdh is used as a reference gene. **f**, **g**, **h** Multiplex immunofluorescence staining for CD4, F4/80, Ly6G, NCR1, and IL-17 at 6 d.p.i. by SARS-CoV-2, alpha or beta variant, and statistics of the percentages of cells positive for CD4, F4/80, Ly6G, NCR1, and IL-17 at 6 d.p.i. (Each dot represents an image, Two-tailed student *t* test, scale bars, 100 μm). **i** Schematic diagram of CD147-CyPA regulation of cytokine expression and blocking effect of Meplazumab

Presently, no specific therapeutic drug has been approved for the treatment of COVID-19. To verify whether CD147 functions as a therapeutic target for COVID-19, we treated SARS-CoV-2, alpha and beta variants infected hCD147 mice with Meplazumab (Supplementary Fig. 7a, c). For SARS-CoV-2 infection, Meplazumab effectively cleared the virus and alveolar exudation, resolving the pneumonia at 6 d.p.i. (Fig. 6a, b and Supplementary Fig. 7b). Strikingly, for alpha and beta variants infection, the virus loading was significantly reduced, and the pneumonia was markedly improved after treatment with Meplazumab at 6 d.p.i. (Fig. 6c, d and Supplementary Fig. 7d). What’s more, either SARS-CoV-2 or it’s variants infection, Meplazumab treatment had a favorable therapeutic effect on the downregulation of cytokines and chemokines at 6 d.p.i., such as IL-6, IL10, IL1b, CCL2, CXCL1, CXCL2, IFN-γ, IL-17, and CyPA (Fig. 6e). Infiltration of macrophages and neutrophils descended after Meplazumab treatment, and importantly, Meplazumab reduced the number of IL-17 positive cells (Fig. 6f–h). Taken together, Meplazumab can competitively block the binding between spike and CD147, thus effectively treat COVID-19 by reducing viral entry and suppressing CyPA-mediated cytokines overexpression (Fig. 6i), supportive of our exploratory phase II clinical trial results.^37^ Therefore, CD147 is a promising target for the treatment of COVID-19 caused by SARS-CoV-2 and its variants.

## Discussion

Since the outbreak of COVID-19, SARS-CoV-2 variants of concern (VOCs) are capturing global attention. The mutation accumulation in human body is driven by the untimely elimination of virions, attributed to continuous virus replication, which is principally due to the untimely clinical treatment and lack of specific and effective drugs. These mutation on VOCs could lead to epitope changes, which reduced the affinity of antibodies administrated by neutralizing antibody therapy or generated by vaccines.^38–40^ Therefore, it is important to develop a universal, specific, and effective drug against VOCs. Our lab recently revealed that CD147 is a receptor mediating cellular entry of SARS-CoV-2.^18^ In our study, we conducted virus inhibition experiments in vitro with CD147 antibody (Meplazumab), which showed that Meplazumab of low concentration effectively blocked the infection and replication of variants—alpha, beta, gamma, and delta at inhibition rates of 75.7, 52.1, 52.1, and 62.3%, respectively. Therefore, Meplazumab can protected host from SARS-CoV-2 infection regardless of mutations identified so far. Therefore, Meplazumab is a promising universal and effective drug against COVID-19.

With the repeated surge of COVID-19, especially the prevalence of VOCs, no ideal mouse model was yet available in evaluating vaccine protection and specific and effective drugs. hACE2 transgenic mouse model has been constructed in 2006,^41^ after outbreak of SARS. Since the COVID-19 epidemic, several vaccines and drugs were evaluated by using the hACE2 mouse model.^42,43^ In 2020, the hACE2 transgenic mouse model with SARS-CoV-2 infection was used to manifest interstitial pneumonia.^44,45^ However, the hACE2 mice cannot fully recapitulate the pathology of severe COVID-19, which features acute exudative alveolar pneumonia. Thus, an infection model that can mimic the typical COVID-19 pneumonia is urgently in need for preclinical studies. In this study, we established a hCD147 transgenic mouse model to investigate the pathogenesis of SARS-CoV-2 infection with CD147. We observed hCD147 mice exhibit the typical exudative alveolar pneumonia of COVID-19 after SARS-CoV-2 infection. When compared with the hACE2 mouse model, SARS-CoV-2-infected hCD147 mice presented stronger inflammatory responses, including activation of the NF-κB pathway, Th17 cell responses, and activation of proinflammatory cytokine and chemokine signaling pathways, which were all reported to be involved in exudative alveolar pneumonia.^27,46,47^ The difference in the phenotype of hACE2 and hCD147 transgenic mice may attribute to their different expression profile. CD147 is expressed on various types of proinflammatory cells including Th17 cells, neutrophils, NK cells,^48,49^ where ACE2 is rarely expressed.^50^ Thus, SARS-CoV-2-infected hCD147 transgenic mice well recapitulate the symptoms of COVID-19 pneumonia, providing a good model for studying the pathogenic mechanism and pharmacodynamics of agents against COVID-19.

Cytokine storm is a main cause of death in severe COVID-19 patients. It was reported that IL-17 is an initiator of cytokine storm during viral infection, which results in tissue damage and ARDS.^27^ The infection of SARS-CoV-2 via CD147 can induce prolonged Th17 cell response, suggesting that CD147 is the key factor to cytokine storm. We found that CyPA, induced by virus infection via CD147 receptor, promotes inflammation by upregulating the expression of IL-17, IL-6, and IL-1β in lung epithelial cells. These results are consistent with published data showing CyPA can promote inflammation in arthritis and pancreatic cancers through the NF-κB pathway and ERK1/2 and MAPK signaling pathways.^51,52^ High level of CyPA not only induces chemotaxis in numerous monocytes and neutrophils to the inflammatory site^53^ but also activates the MAPK signaling pathway to induce more cytokines and chemokines expression. Our results showed that targeting CD147 can effectively inhibit the proinflammatory effects of CyPA, indicating that CD147 could be a therapeutic target for SARS-CoV-2-related cytokine storms. Considering the two important receptors CD147 and ACE2 for viral infection, it is possible that the combination of targeting both the CD147 and ACE2 receptors might play a synergistic effect on inhibiting virus infection.

Taken together, CD147 plays a central role in the infection of SARS-CoV-2 and its variants and pathogenesis of COVID-19. CD147 is not only a universal viral entry receptor for SARS-CoV-2 and its four prevalent variants—alpha, beta, gamma, delta but also a signal initiator for cytokine storm. CD147 antibody Meplazumab inhibits SARS-CoV-2 and variants infection and suppresses cytokine storm by blocking the direct interaction between CD147 and spike protein. Our findings provide a new perspective for pathogenesis of COVID-19 and a critical target for VOCs in COVID-19 treatment.

## Materials and methods

### Virus and cell lines

The SARS-CoV-2, alpha, beta, gamma, and delta variants strains used for in vitro experiments was obtained from the State Key Laboratory of Pathogen and Biosecurity at Beijing Institute of Microbiology and Epidemiology. The SARS-CoV-2, alpha and beta variants strains used for in vivo experiments was from the Chinese Academy of Medical Sciences.

The VeroE6 and BEAS-2B cell lines were obtained from the Cell Bank of the Chinese Academy of Sciences (Shanghai, China). All cell lines were authenticated using short tandem repeat DNA profiling at Beijing Microread Genetics Co., Ltd. (Beijing, China) and cultured at 37 °C under 5% CO_2_ in Dulbecco’s modified Eagle’s medium (DMEM, Invitrogen) or RPMI 1640 medium supplemented with 10% fetal bovine serum (FBS, Life Technologies), 1% penicillin/streptomycin, and 2% L-glutamine.

### hACE2 and hCD147 mice

Human CD147 transgenic mice (hCD147) were provided by the Shanghai Model Organisms Center, Inc. (China). The extracellular region of CD147 in wild-type (WT) C57BL/6 J mice was replaced with human CD147 by targeting embryonic stem cells. Human ACE2 transgenic mice (hACE2) were provided by the National Institutes for Food and Drug Control. These mice were bred and maintained in a specific pathogen-free facility at the Chinese Academy of Medical Sciences. The animal experiments were performed in accordance with the People’s Republic of China Legislation Regarding the Use and Care of Laboratory Animals. All protocols used in this study were approved by the Institutional Animal Care and Use Committee of Fourth Military Medical University (20200206).

### Plasma and lung tissue sections from COVID-19 patients

Plasma was obtained from healthy individuals (*n* = 100) at Xijing Hospital of the Fourth Military Medical University, and plasma was obtained from COVID-19 patients (*n* = 206) at Zhongnan Hospital of Wuhan University. Lung tissue sections were obtained from COVID-19 patients at Tangdu Hospital of the Fourth Military Medical University (*n* = 2) and the Institute of Clinical Pathology of the Third Military Medical University (*n* = 11).

This study was approved by the ethics committees of Xijing Hospital and Tangdu Hospital, Fourth Military Medical University (KY20202005-1, K202002-01, E202003-01), Third Military Medical University (2020074), and Zhongnan Hospital of Wuhan University (2020056 K).

### Generation of stable knockdown and knockout cell lines

VeroE6 cells were transfected with supernatants containing lentiviruses encoding the shCD147 and shACE2 constructs (GeneChem Co. Ltd.) using Lipofectamine 2000 reagent (Invitrogen) to generate the CD147 and ACE2 knockdown cell lines, respectively. After 48 h, the infected cells were selected with 3 μg/ml puromycin, and monoclonal cells were selected for further study. CD147−/−ACE2−/− VeroE6 cells were generated using the CRISPR/Cas9 system (GeneChem Co. Ltd).

### Infection of hCD147 and hACE2 mice with SARS-CoV-2

After being anesthetized, each mouse was intranasally inoculated with SARS-CoV-2 at a TCID_50_ of 3 × 10^5^. For CD147 antibody treatment, 3 mg/kg CD147 antibody (Meplazumab) was administered via the tail vein at 1 d.p.i. Lung tissues were collected for RNA extraction or fixed with paraformaldehyde and PLP Fixing Solution for H&E staining, IHC, immunofluorescence staining, and TEM.

### Histological analysis

Mouse lung samples were fixed with 4% paraformaldehyde, embedded in paraffin and cut into 3-µm-thick sections. Formalin-fixed paraffin-embedded mouse lung tissue sections were deparaffinized with xylene and alcohol. Fixed tissue samples were used for hematoxylin-eosin (H&E) staining and indirect IHC. For H&E staining, the slides were counterstained with hematoxylin for 15 min and eosin for 10 min. For IHC, the slides were deparaffinized and rehydrated, followed by 15 min of blocking with 5% goat serum in phosphate-buffered saline (PBS) and staining with primary antibodies for 2 h. Immunoperoxidase staining was performed using a General SP kit (SP-9000, ZSGB-BIO), and the sections were then treated with 3,3’-diaminobenzidine (ZLI-9019, ZSGB-BIO) to detect the target proteins, followed by counterstaining of nuclei with hematoxylin.

### Multiplex immunofluorescence staining

Multiplex immunofluorescence analyses were performed using 3-μm-thick sections of formalin-fixed and paraffin-embedded tissues. Briefly, slides were deparaffinized in xylene and hydrated in ethanol at gradient concentrations. After heat-induced antigen retrieval in either citrate (pH = 6) or Tris-EDTA (pH = 9) buffer, the samples were permeabilized with 0.5% Triton X-100, blocked with 5% goat serum-PBS, and stained with primary antibodies. Nuclei were stained with DAPI. A TSA-indirect kit was used according to the manufacturer’s instructions (PerkinElmer). Image analysis was performed using HALO software (Akoya Biosciences).

### Quantitative measurement of cytokines

Forty human cytokines were detected by Quantibody® array kits (QAH-INF-3, RayBiotech) according to the manufacturer’s protocol. Then, 100 µl of standard cytokines or samples was added to each well after blocking, followed by incubation with the arrays at room temperature for 2 h. The wells were washed, and 80 µl of the detection antibody cocktail was added to each well and incubated at room temperature for 1 h. Eighty microlitres of Cy3 equivalent dye-conjugated streptavidin was added to each well and incubated in the dark at room temperature for 1 h. The signals were visualized by Axon GenePix. The data were analysed by microarray analysis software (GenePix, ScanArray Express, ArrayVision and MicroVigene).

### Transmission electron microscopy (TEM)

The lung tissues were cut into 1 mm^3^ pieces and fixed with PLP Fixing Solution (G2220, Solarbio) for at least 24 h. After washing with PBS, the sections were treated with osmic acid for 1.5 h. Then, the tissues were dehydrated with alcohol at gradient concentrations and soaked in acetone for 15 min. After embedding and polymerization overnight with epoxy resin, the slices were cut with an ultrathin slicer and glued to a perforated copper grid. Finally, each section was stained with lead citrate and uranium solution for 10 min, washed with water, and dried. The ultrastructure was observed by transmission electron microscopy (JEM-1230, JEOLLTD).

### In vitro virus infection test

The cells were cultured at 37 °C under 5% CO_2_ overnight, and the cell medium was then replaced by a medium containing the virus. After the cells were infected for 1 h at 37 °C, the virus supernatant was discarded, and the cells were washed twice with PBS. Finally, the cells were cultured with 2% FBS maintenance medium with or without CD147 antibody (Meplazumab, 15 or 60 μg/ml). At 48 h after infection, viral and cellular RNA were extracted together and detected by RT-qPCR.

### In vitro SARS-CoV-2 pseudovirus infection test

The SARS-CoV-2 pseudovirus and its variants expressing luciferase were obtained from the Institute for Biological Product Control, National Institutes for Food and Drug Control (Beijing, China). The SARS-CoV-2 pseudovirus was added to VeroE6 cells at a TCID_50_ of 1300 either with or without 60 μg/ml CD147 antibody. Meplazumab was humanized CD147 monoclonal antibodies, produced by Jiangsu Pacific Meinuoke Biopharmceutical Co. Ltd. Human IgG was used as a control. The luciferase signal was detected using a Dual-Luciferase Reporter Assay System (E1960, Promega) according to the manufacturer’s protocol.

### Statistical analysis

The cytokines detected by the protein chip were analysed by moderated *t*-statistics. ELISA data were measured using a parameter logistic curve. All multiplex fluorescence and immunohistochemical staining images were analysed by HALO Image Analysis Software, and significant differences were calculated by unpaired *t* tests. Correlations were analysed by Pearson correlation coefficients. Significant differences in cell and mouse tests were analysed by unpaired *t* tests with a two-tailed distribution. *p* < 0.05 was considered to be statistically significant. Statistical analyses were performed using GraphPad Prism, version 8.0.

## Supplementary information

Supplementary_Materials and figures

## Data Availability

The data that support the findings of this study are available from the corresponding author upon request (znchen@fmmu.edu.cn) and has been uploaded to GEO (GSE167403). All data are available in the main text or the Supplementary materials.
