# Supplementary material for: CD147 antibody specifically and effectively inhibits infection and cytokine storm of SARS-CoV-2 and its variants delta, alpha, beta, and gamma
Source: Signal Transduct Target Ther. 2021 Sep 25;6:347. doi: 10.1038/s41392-021-00760-8 (PMC8464593; doi:10.1038/s41392-021-00760-8)
Supplement: Supplementary file 1 — Supplementary_Materials and figures [file 41392_2021_760_MOESM1_ESM.docx]

Supplementary Materials for

**CD147 antibody specifically and effectively inhibits infection and cytokine storm of SARS-CoV-2 and its variants delta, alpha, beta and gamma**

Jiejie Geng^1†^, Liang Chen^2†^, Yufeng Yuan^4†^, Ke Wang^1†^, Youchun Wang^5†^, Chuan Qin^6†^, Guizhen Wu^7†^, Ruo Chen^1^, Zheng Zhang^1^, Ding Wei^1^, Peng Du^8^, Jun Zhang^8^, Peng Lin^1^, Kui Zhang^3^, Yongqiang Deng^9^, Ke Xu^7^, Jiangning Liu^6^, Xiuxuan Sun^1^, Ting Guo^1^, Xu Yang^1^, Jiao Wu^1^, Jianli Jiang^1^, Ling Li^1^, Kun Zhang^1^, Zhe Wang^10^, Jing Zhang^10^, Qingguo Yan^10^, Hua Zhu^6^, Zhaohui Zheng^3^, Jinlin Miao^1^, Xianghui Fu^3^, Fengfan Yang^3^, Xiaochun Chen^11^, Hao Tang^11^, Yang Zhang^1^, Ying Shi^1^, Yumeng Zhu^1^, Zhuo Pei^1^, Fei Huo^1^, Xue Liang^1^, Yatao Wang^1^, Qingyi Wang^10^, Wen Xie^4^, Yirong Li^4^, Mingyan Shi^1^, Huijie Bian^1*^, Ping Zhu^3*^, Zhi-Nan Chen^1*^

Correspondence to: [znchen@f](mailto:xxxxx@xxxx.xxx)mmu.edu.cn

**This PDF file includes:**

Materials and Methods

Figures. S1 to S7

**Materials and Methods**

**Enzyme-linked immunosorbent assay (ELISA)**

The content of CyPA in plasma was detected using ELISA kits according to the manufacturer’s instructions (Eiaab Science, Inc., 0979 h). In brief, the microtiter plate provided in the kit was precoated with a biotin-conjugated antibody specific to the target CyPA. Standards were established at 6 different dilutions ranging from 0 ng/ml to 10 ng/ml, and nonimmune serum was used as a negative control. Next, 100 µl of the plasma, standard or negative control was added to each well of the plate and incubated for 2 h at 37°C. Then, avidin conjugated to horseradish peroxidase (HRP) was added to each well and incubated. The reaction was developed by adding 100 µl of 3,3',5,5'-tetramethylbenzidine (TMB) to the wells for 12 min at room temperature. Finally, 200 M sulfuric acid was added to stop the reaction, and the absorbance at 450 nm was determined using a BioTeck Epoch microplate reader.

**RNA-seq**

Total RNA was extracted using Trizol reagent kit (Invitrogen, Carlsbad, CA,USA) according to the manufacturer’s protocol. RNA quality was assessed on an Agilent 2100 Bioanalyzer (Agilent Technologies, Palo Alto, CA, USA) and checked using RNase free agarose gel electrophoresis. After total RNA was extracted, eukaryotic mRNA was enriched by Oligo (dT) beads, while prokaryotic mRNA was enriched by removing rRNA by Ribo-ZeroTM Magnetic Kit (Epicentre, Madison, WI, USA). Then the enriched mRNA was fragmented into short fragments using fragmentation buffer and reverse transcripted into cDNA with random primers. Second-strand cDNA were synthesized by DNA polymerase I, RNase H, dNTP and buffer. Then the cDNA fragments were purified with QiaQuick PCR extraction kit (Qiagen, Venlo, The Netherlands), end repaired, poly(A) added, and ligated to Illumina sequencing adapters. The ligation products were size selected by agarose gel electrophoresis, PCR amplified, and sequenced using Illumina Novaseq6000 by Gene Denovo Biotechnology Co. (Guangzhou, China). The datasets generated in the current study are available from the corresponding author upon reasonable request.

**RNA extraction and real-time quantitative PCR analysis**

Total RNA was extracted using Total RNA Kit II (Omega Bio-tek) according to the manufacturer’s instructions. cDNA was synthesized from 1 μg of total RNA using an RNA reverse transcriptase kit (Takara). Real-time qPCR was performed using the ABI PRISM 7000 Sequence Detection System (Applied Biosystems), and SYBR Premix Ex Taq II (Takara) was used for amplification according to the manufacturer’s instructions. The cDNA inputs were standardized, and PCR was performed for 40 cycles. The mouse organ tissues were homogenized to detect the virus gene copy number by qPCR (TaqMan Universal Master Mix II with UNG, 4440044, Applied Biosystems). The sequences of the primers are listed in below table:

**The primers for qPCR**

| **Gene name** | **Forward primer (5’ - 3)** | **Reverse primer (5’ - 3’)** |
| --- | --- | --- |
| SARS-CoV-2 N protein | GGGGAACTTCTCCTGCTAGAAT | CAGACATTTTGCTCTCAAGCTG |
| SARS-CoV-2 N protein probe | FAM-TTGCTGCTGCTTGACAGATT-BHQ1 | |
| Mouse IL-6 | GACTGGGGATGTCTGTAGCTC | CAACTGGATGGAAGTCTCTTGC |
| Mouse IL8 | TAGGCATCTTCGTCCGTCC | CCAACAGTAGCCTTCACCCAT |
| Mouse CCL2 | CACTCACCTGCTGCTACTCA | TGAGCTTGGTGACAAAAACTACAG |
| Mouse IL1b | TGCCACCTTTTGACAGTGATG | AAGGTCCACGGGAAAGACAC |
| Mouse IL10 | GGTTGCCAAGCCTTATCGGA | CACCTTGGTCTTGGAGCTTATT |
| Mouse IL-17 | CCTGGACTCTCCACCGCAA | CGACCCTGAAAGTGAAGGGG |
| Mouse CCL8 | TACGCAGTGCTTCTTTGCCT | TGCTTGGTCTGGAAAACCACA |
| Mouse CCL3 | CAGCCAGGTGTCATTTTCCTG | CTCGATGTGGCTACTTGGCA |
| Mouse CXCL1 | ACCCAAACCGAAGTCATAGCC | TTGTCAGAAGCCAGCGTTCA |
| Mouse CXCL2 | CCCAGACAGAAGTCATAGCCAC | CGAGGCACATCAGGTACGAT |
| Mouse CXCL9 | CAGTGTGGAGTTCGAGGAACC | AGGCAGGTTTGATCTCCGTT |
| Mouse CXCL10 | ACGTGTTGAGATCATTGCCAC | CCATCCACTGGGTAAAGGGG |
| Mouse IFNγ | GAACTGGCAAAAGGATGGTG | GTTGCTGATGGCCTGATTGTC |
| Mouse TNFα | GTCCCCAAAGGGATGAGAAGT | TTTGCTACGACGTGGGCTAC |
| Mouse CyPA | CGTGTTCTTCGACATTG | CCTGACACATAAACCCT |
| Mouse GAPDH | CAGTGGCAAAGTGGAGATTGTTG | TCGCTCCTGGAAGATGGTGAT |
| Human CCL2 | ATCACCAGCAGCAAGTGTCC | TGGGTTGTGGAGTGAGTGTT |
| Human IL-6 | TCTCCACAAACATGTAACAAGAGT | CACAGCTCTGGCTTGTTCCT |
| Human GAPDH | GCACCGTCAAGGCTGAGAAC | GCACCGTCAAGGCTGAGAAC |
| VeroE6 CCL2 | TCTGTGCCTGCTGCTCATAG | CAGATCTCCTTGGCCACAA |
| VeroE6 IL-6 | AGGAGACTTGCCTGGTGAAA | CAGGGGTGGTTATTGCATCT |
| VeroE6 CyPA | CGTGTTCTTCGACATTG | CCTGACACATAAACCCT |
| VeroE6 GAPDH | GCACCGTCAAGGCTGAGAAC | TGGTGAAGACGCCAGTGGA |

**Surface plasmon resonance (SPR)**

SPR analysis was performed on a Biacore 3000 system (Biacore). In brief, His-CyPA (produced by our laboratory) was fixed to the surface of CM5 sensor chips (GE Healthcare Bio-Sciences AB) by an Amine Coupling Kit (GE Healthcare, BR-1000-50). The interaction between CyPA and CD147 was detected using the kinetic analysis/concentration series/direct binding mode; the flow rate was set to 15 μl/min, and the binding and dissociation time was 3 min. The results were analysed by BIAevaluation software (Biacore) to determine the affinity constants.

**Co-immunoprecipitation (Co-IP)**

Co-IP assays were performed using a Pierce® Co-Immunoprecipitation Kit (26149, Thermo Fisher Scientific, Inc.) according to the manufacturer's protocol. A mouse anti-human CyPA antibody (50 μg) and mouse anti-human CD147 antibody (Jiangsu Pacific Meinuoke Biopharmaceutical Co. Ltd, China, 50 μg) were used for antibody immobilization. The eluted proteins were detected by Western blot. Having boiled for 5 min, the eluted proteins were loaded onto a 12% SDS-PAGE gel and then transferred to a PVDF membrane (Millipore). After blocking with 5% nonfat milk for 1 h, the membrane was incubated with the corresponding primary antibodies at 4°C overnight. The images were developed following incubation with the secondary antibody (goat anti-mouse IgG (H+L), 31430, Thermo Fisher Scientific, 1:5000 dilution) at room temperature for 1 h.

Figure. S1.


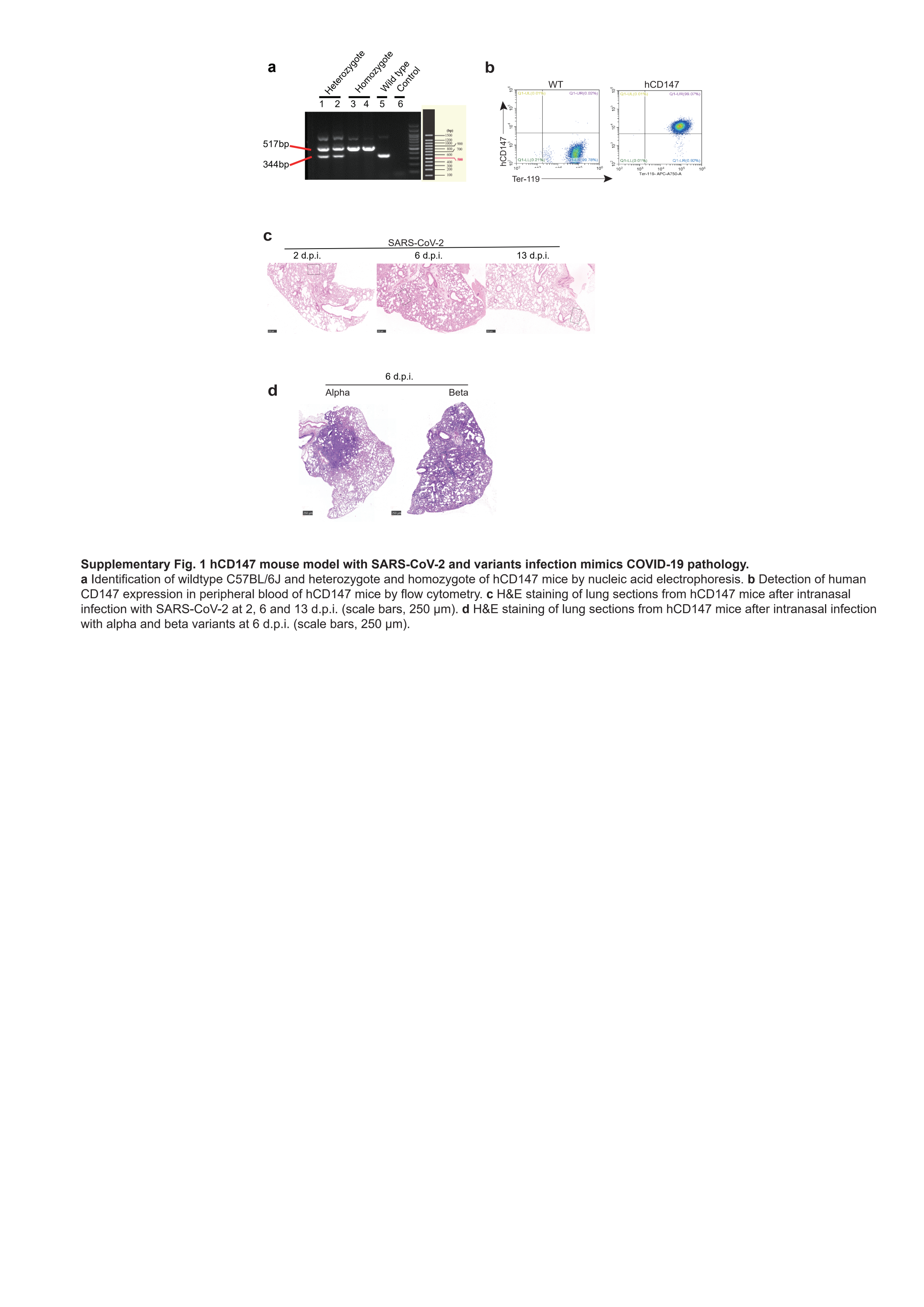


Supplementary Fig. 1 hCD147 mouse model with SARS-CoV-2 and variants infection mimics COVID-19 pathology. a Identification of wildtype C57BL/6J and heterozygote and homozygote of hCD147 mice by nucleic acid electrophoresis. b Detection of human CD147 expression in peripheral blood of hCD147 mice by flow cytometry. c H&E staining of lung sections from hCD147 mice after intranasal infection with SARS-CoV-2 at 2, 6 and 13 d.p.i. (scale bars, 250 μm). d H&E staining of lung sections from hCD147 mice after intranasal infection with alpha and beta variants at 6 d.p.i. (scale bars, 250 μm).

Figure. S2.


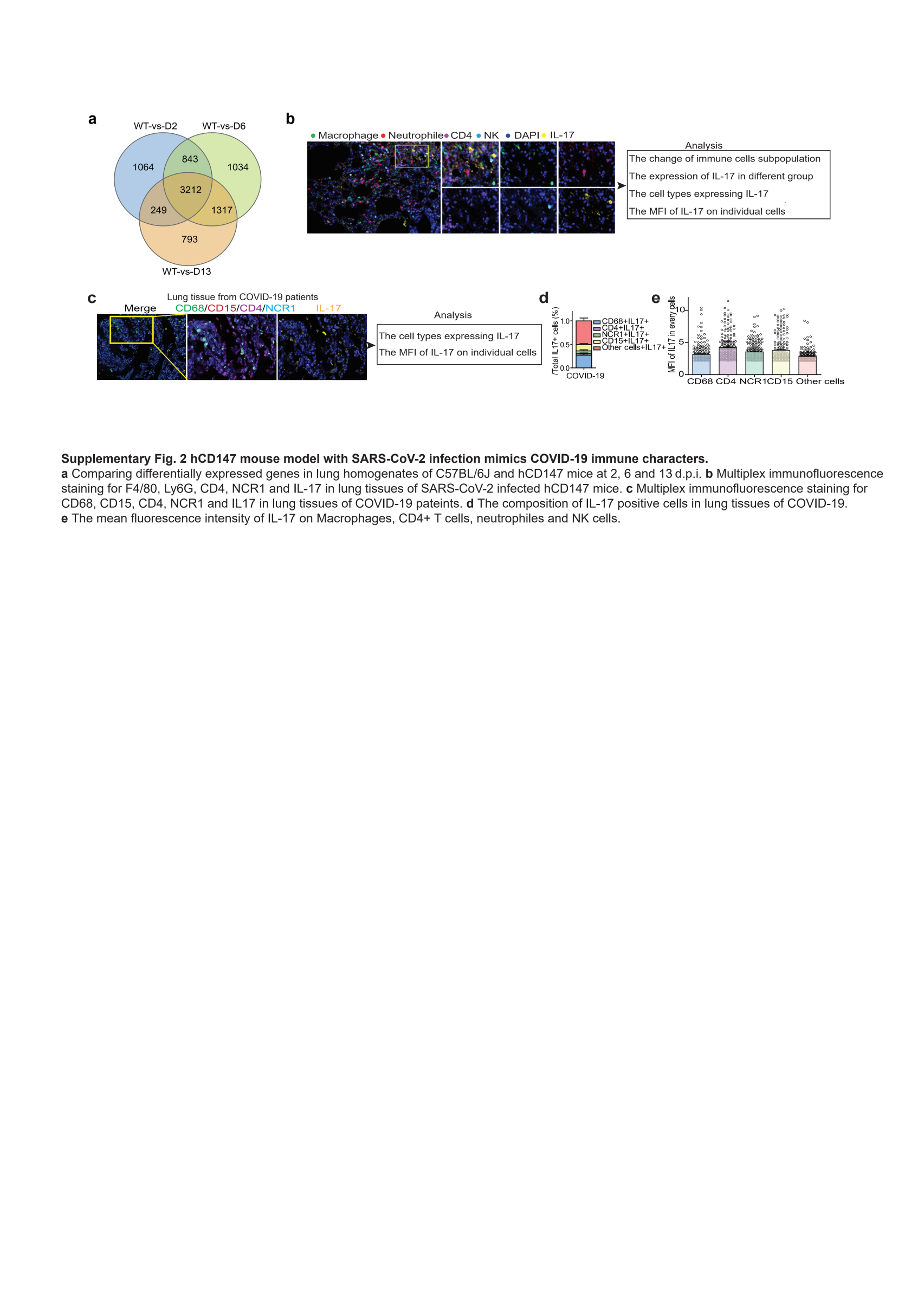


**Supplementary Fig. 2 hCD147 mouse model with SARS-CoV-2 infection mimics COVID-19 immune characters. a** Comparing differentially expressed genes in lung homogenates of C57BL/6J and hCD147 mice at 2, 6 and 13 d.p.i. **b** Multiplex immunofluorescence staining for F4/80, Ly6G, CD4, NCR1 and IL-17 in lung tissues of SARS-CoV-2 infected hCD147 mice. **c** Multiplex immunofluorescence staining for CD68, CD15, CD4, NCR1 and IL17 in lung tissues of COVID-19 pateints. **d** The composition of IL-17 positive cells in lung tissues of COVID-19. **e** The mean fluorescence intensity of IL-17 on Macrophages, CD4+ T cells, neutrophiles and NK cells.

Figure. S3.


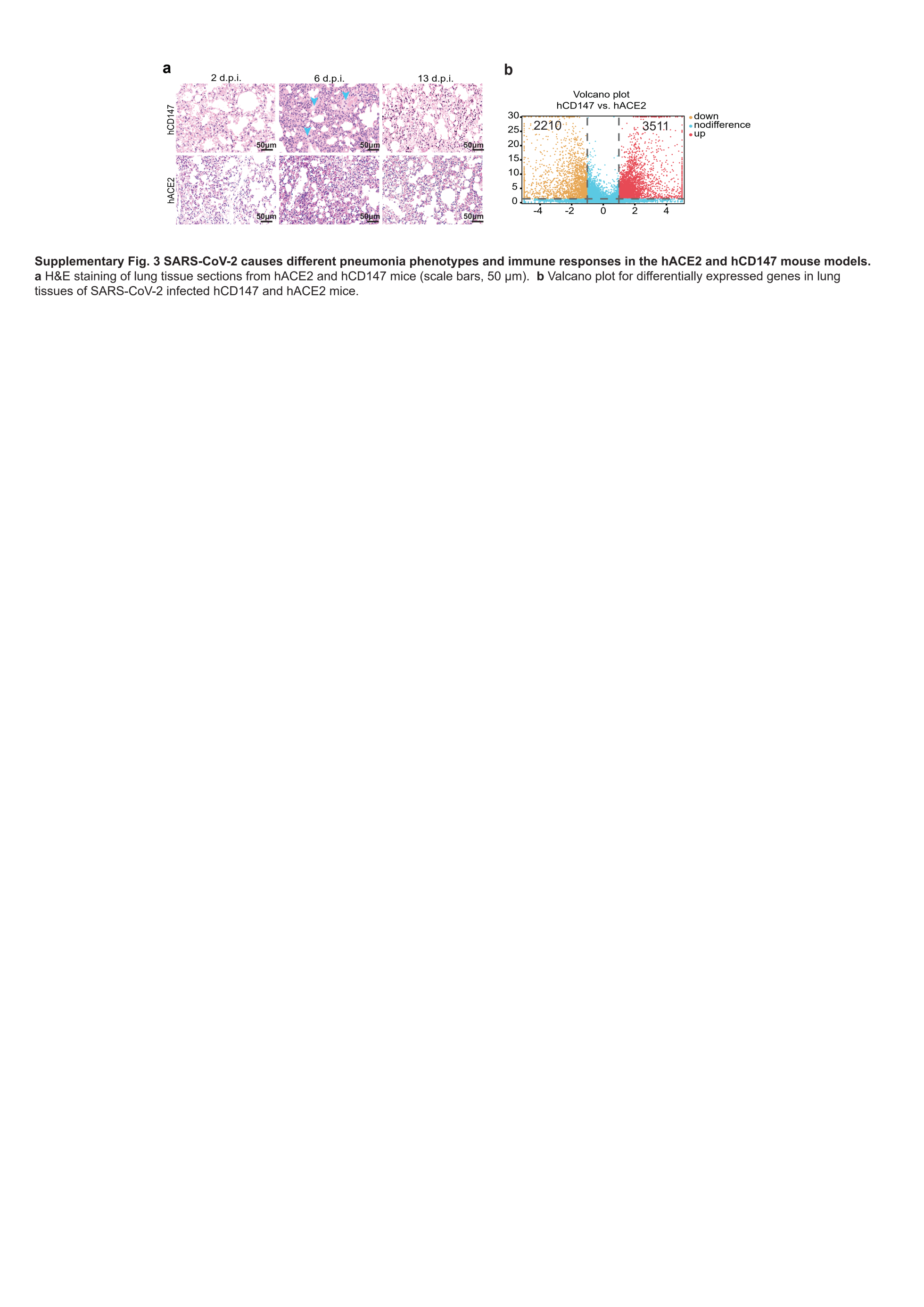


**Supplementary Fig. 3 SARS-CoV-2 causes different pneumonia phenotypes and immune responses in the hACE2 and hCD147 mouse models. a** H&E staining of lung tissue sections from hACE2 and hCD147 mice (scale bars, 50 μm). **b** Valcano plot for differentially expressed genes in lung tissues of SARS-CoV-2 infected hCD147 and hACE2 mice.

Figure. S4.


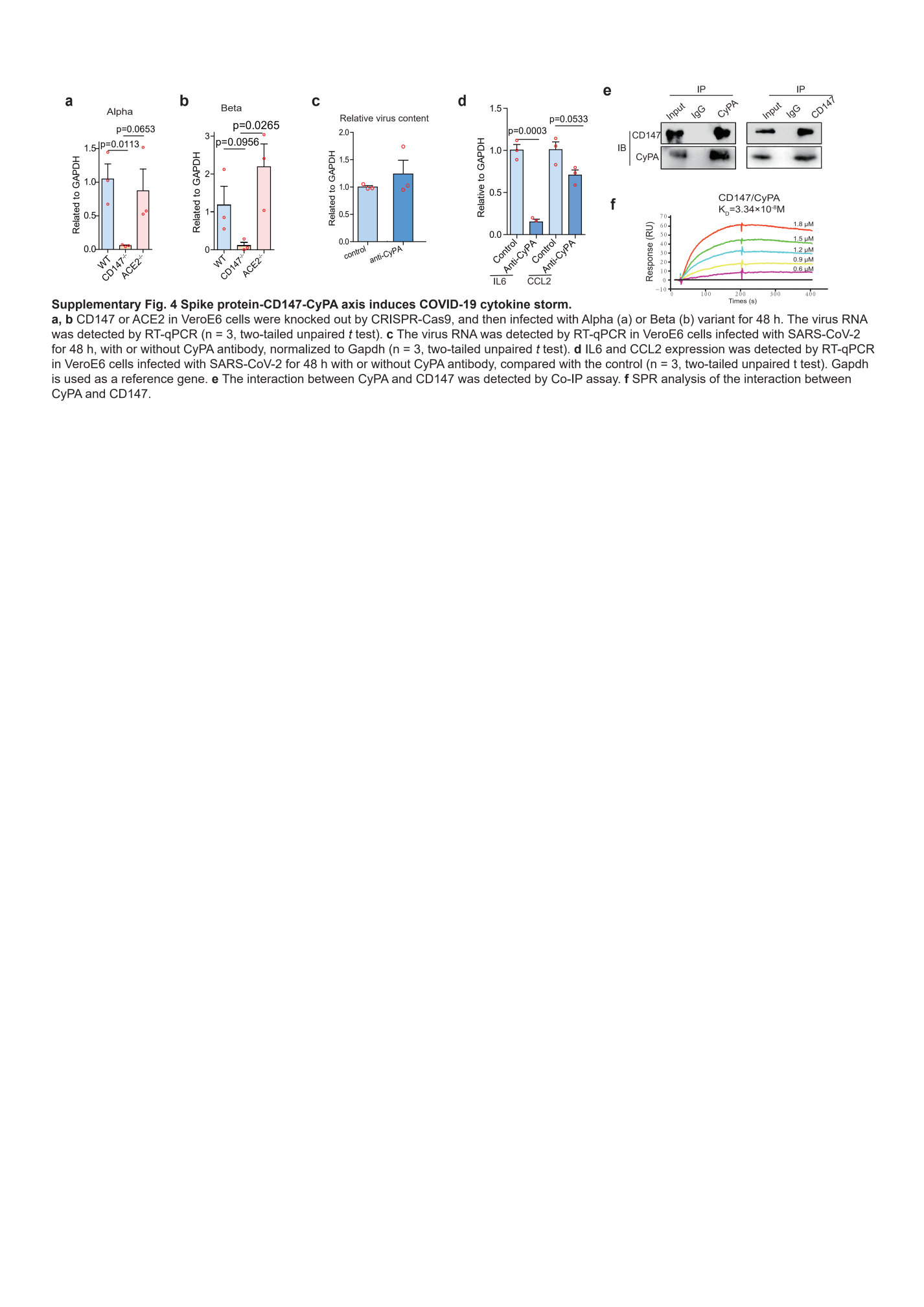


**Supplementary Fig. 4 Spike protein-CD147-CyPA axis induces COVID-19 cytokine storm. a, b** CD147 or ACE2 in VeroE6 cells were knocked out by CRISPR-Cas9, and then infected with Alpha (a) or Beta (b) variant for 48 h. The virus RNA was detected by RT-qPCR (n = 3, two-tailed unpaired t test). **c** The virus RNA was detected by RT-qPCR in VeroE6 cells infected with SARS-CoV-2 for 48 h, with or without CyPA antibody, normalized to Gapdh (n = 3, two-tailed unpaired t test). **d** IL6 and CCL2 expression was detected by RT-qPCR in VeroE6 cells infected with SARS-CoV-2 for 48 h with or without CyPA antibody, compared with the control (n = 3, two-tailed unpaired t test). Gapdh is used as a reference gene. **e** The interaction between CyPA and CD147 was detected by Co-IP assay. **f** SPR analysis of the interaction between CyPA and CD147.

Figure. S5.


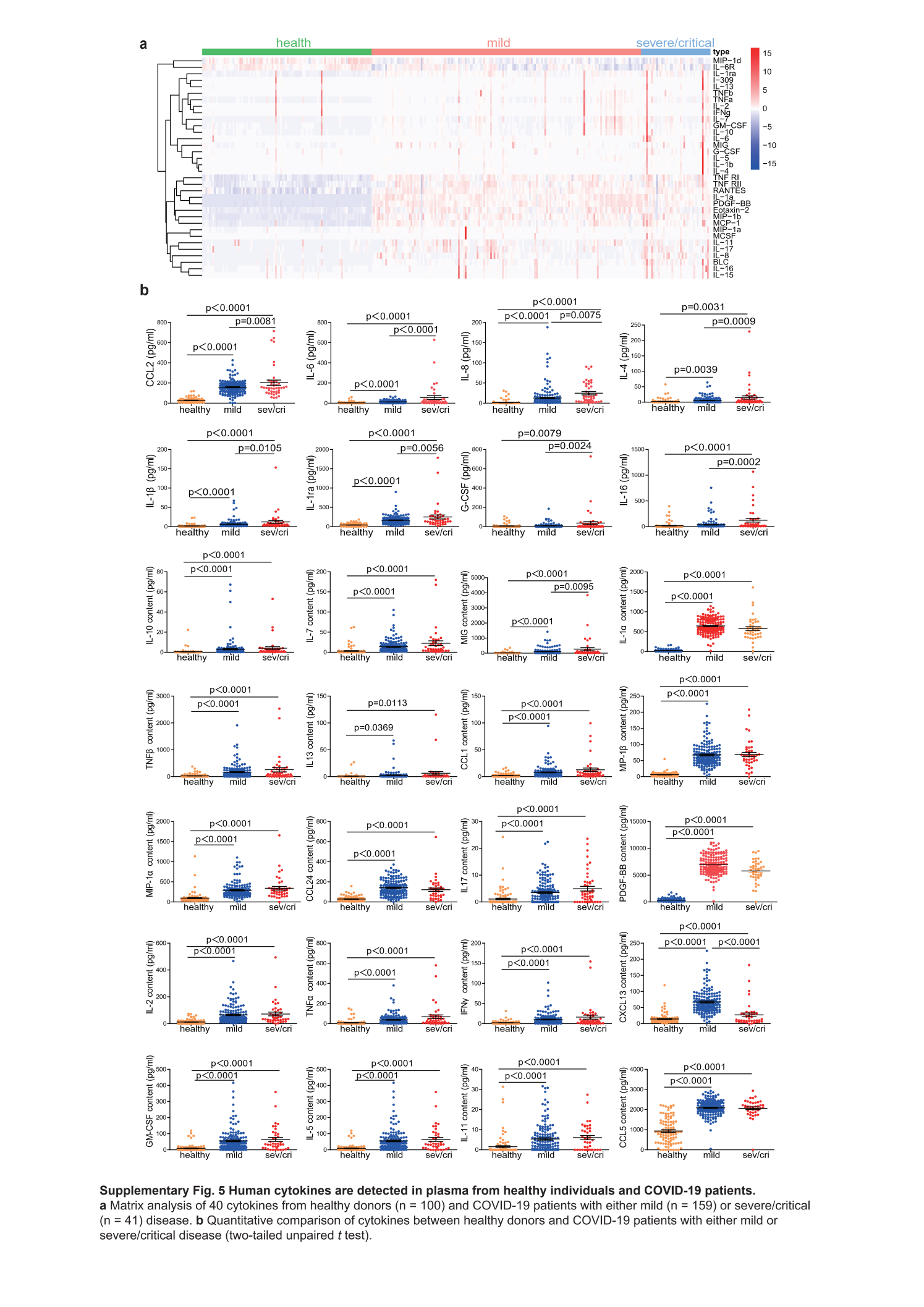


**Supplementary Fig. 5 Human cytokines are detected in plasma from healthy individuals and COVID-19 patients. a** Matrix analysis of 40 cytokines from healthy donors (n = 100) and COVID-19 patients with either mild (n = 159) or severe/critical (n = 41) disease. **b** Quantitative comparison of cytokines between healthy donors and COVID-19 patients with either mild or severe/critical disease (two-tailed unpaired *t* test).

Figure. S6.


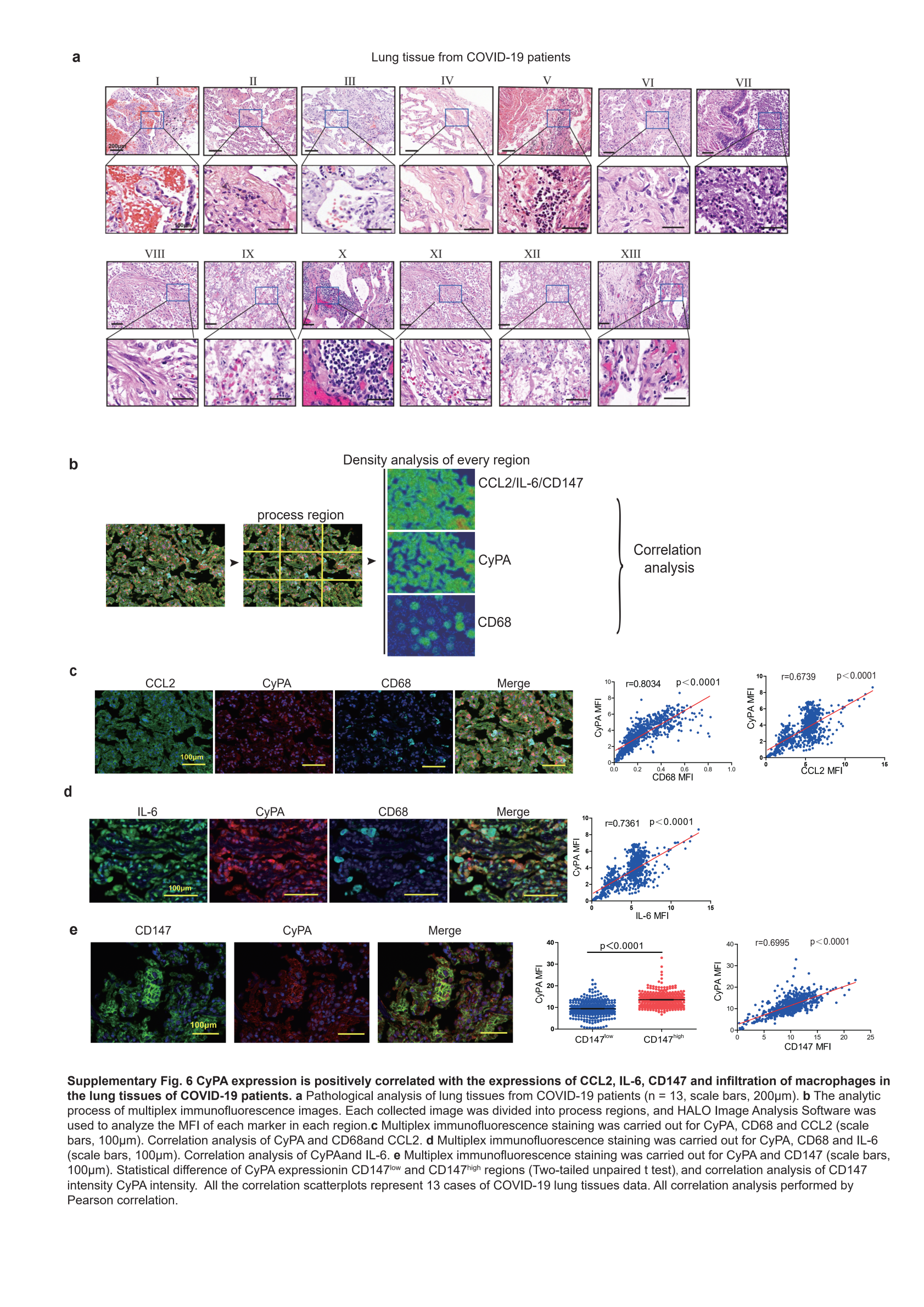


**Supplementary Fig. 6 CyPA expression is positively correlated with the expressions of CCL2, IL-6, CD147 and infiltration of macrophages in the lung tissues of COVID-19 patients. a** Pathological analysis of lung tissues from COVID-19 patients (n = 13, scale bars, 200μm). **b** The analytic process of multiplex immunofluorescence images. Each collected image was divided into process regions, and HALO Image Analysis Software was used to analyze the MFI of each marker in each region.**c** Multiplex immunofluorescence staining was carried out for CyPA, CD68 and CCL2 (scale bars, 100μm). Correlation analysis of CyPA and CD68and CCL2. **d** Multiplex immunofluorescence staining was carried out for CyPA, CD68 and IL-6 (scale bars, 100μm). Correlation analysis of CyPAand IL-6. **e** Multiplex immunofluorescence staining was carried out for CyPA and CD147 (scale bars, 100μm). Statistical difference of CyPA expressionin CD147low and CD147high regions (Two-tailed unpaired t test), and correlation analysis of CD147 intensity CyPA intensity. All the correlation scatterplots represent 13 cases of COVID-19 lung tissues data. All correlation analysis performed by Pearson correlation.

Figure. S7.


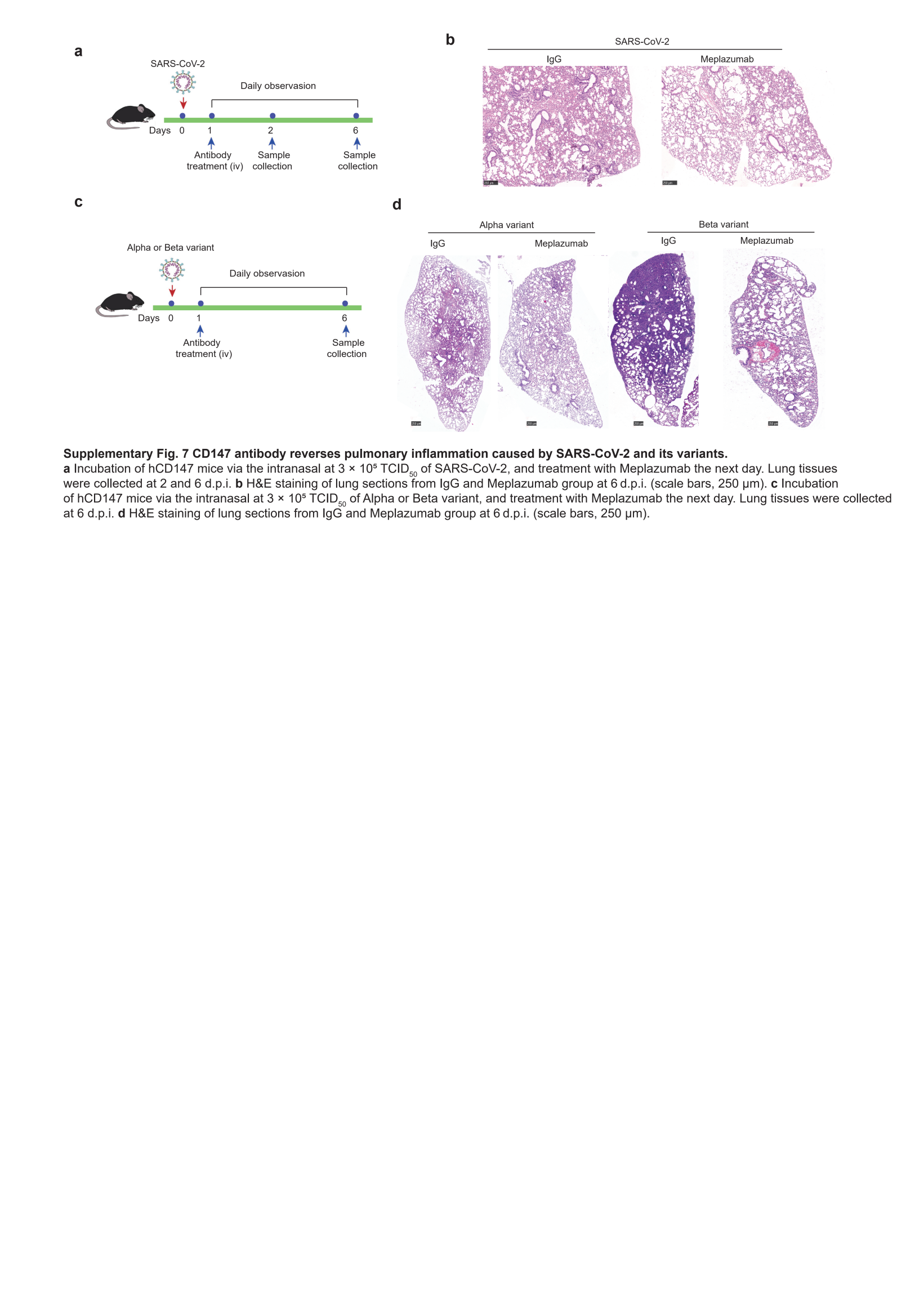


**Supplementary Fig. 7 CD147 antibody reverses pulmonary inflammation caused by SARS-CoV-2 and its variants. a** Incubation of hCD147 mice via the intranasal at 3×10⁵ TCID_50_ of SARS-CoV-2, and treatment with Meplazumab the next day. Lung tissues were collected at 2 and 6 d.p.i. **b** H&E staining of lung sections from IgG and Meplazumab group at 6 d.p.i. (scale bars, 250 μm). **c** Incubation of hCD147 mice via the intranasal at 3×10⁵ TCID_50_ of Alpha or Beta variant, and treatment with Meplazumab the next day. Lung tissues were collected at 6 d.p.i. **d** H&E staining of lung sections from IgG and Meplazumab group at 6 d.p.i. (scale bars, 250 μm).
